# Evaluating public opinions: informing public health policy adaptations in China amid the COVID-19 pandemic

**DOI:** 10.1038/s41598-024-55684-4

**Published:** 2024-03-01

**Authors:** Chenyang Wang, Xinzhi Wang, Pei Wang, Qing Deng, Yi Liu, Hui Zhang

**Affiliations:** 1 Institute of Public Safety Research, Department of Engineering Physics, Tsinghua University, Beijing, 100084 People’s Republic of China; 2 Institute of Public Safety Research, Tsinghua University, Beijing, 100084 People’s Republic of China; 3 School of Computer Engineering and Science, Shanghai University, Shanghai, 200444 People’s Republic of China; 4 Research Institute of Macro-Safety Science, University of Science and Technology Beijing, Beijing, 100083 People’s Republic of China; 5 Public Order School, People’s Public Security University of China, Beijing, 100872 People’s Republic of China

**Keywords:** Public opinion, Policy informatics, Public health, Social media, Applied mathematics, Scientific data, Statistics, Risk factors

## Abstract

Public concern regarding safety policies serious consequences is anticipated to persist over an extended duration. A study examining a case of rapid public health policy adaptation in China during the COVID-19 epidemic was conducted by gathering public opinion data from major social media platforms. A systematic approach to comprehend public opinion was developed. Five fundamental elements and four dimensions were delineated. An indicator system was established utilizing the K-means text clustering model. Public prediction, expectation, and their evolution underlying public concern were elucidated employing TF–IDF text mining models. The HMM elucidated the way public opinion influences policy adjustments. The findings underscore that public concern regarding enduring events undergoes temporal shifts, mirroring the evolution of public opinion towards policy. Public opinion aroused by both the original event and derived events collaboratively influence policy adjustments. In China, public opinion serves as a mechanism for policy feedback and oversight; notably, negative public sentiment plays a pivotal role in expediting policy transitions. These findings aid in refining policies to mitigate emergencies through a feedback loop, thereby averting the emergence of safety risks such as social unrest prompted by public opinion.

## Introduction

Public opinion has been recognized as an important resource for political decision-making and adjustment in democracies^1^, but there has been no clear evidence of the role it plays in other types of governments. For example, in China, with the governing system of the people’s democratic dictatorship, public opinion has been considered to play a policy monitoring role in previous studies^2^. However, public opinion in China plays an increasingly strong role in policy adjustment as society develops^3^. Our study provides evidence of this change by examining public opinion data related to abrupt public health policy changes^4^ during the COVID-19 epidemic in China.

Public attention increases during public emergency events^5^. Public emergency events can be classified as short-term^6^ and long-term based on their duration from initiation to conclusion^7^. Short-term events encompass occurrences such as earthquakes and fires, while long-term events encompass phenomena such as epidemics. The level of public attention given to public emergency events changes as events unfold^8^, especially during long-term events. The COVID-19 pandemic, regarded as a long-term event, spread widely around the world and caused significant economic damage^9,10^. It also had serious consequences, such as death^11^, fear^12^, and medical resource exhaustion, which affected normal aspects of life for residents, such as travel^13^, work, school^14^, social, and home life^15^. Moreover, public sentiments and viewpoints regarding these occurrences evolve in response to alterations in the spatial and temporal ramifications of event outcomes, as well as their relevance to public life^16^. Throughout the evolution of long-term events, the sudden emergence of public incidents linked to both the initial emergency and adjustments in policies can act as triggers for public deliberation^17^. The transmission characteristics of the different COVID-19 strains vary^18^, and countries offered different response policies considering the national environment and dynamically adjusted these policies as the epidemic evolved^19^, which has sparked wide-ranging social debate. In particular, China has experienced dramatic changes in its epidemiological situation and policies since November 2022, during which time the public has engaged in intense discussion on social media^20^.

This dynamic process is further complicated by the uncertainty surrounding the progression of the event and the potential adaptation of policies in directions perceived as unfavorable to the public, which can collectively evoke negative emotions such as anxiety and anger under public attention^21^. Research has explored the accumulation of adverse public sentiments in the context of public emergencies, revealing that under prolonged public health events, anger tends to accumulate more prominently among the public^22^. While authoritative statements have the capacity to assuage public anxiety, addressing anger is a more intricate process. One can speculate that anger accrues as the event unfolds, necessitating a form of release.

Social media platforms provide channels for internet users to access information and express their views^23^. The platforms commonly used by Chinese netizens include Sina Weibo, official WeChat accounts^24^, and Baidu push notifications^25,26^. Each platform has a different focus. For example, Sina Weibo updates and delivers news every 10 minutes. Users can browse topics on Weibo and click on related topics to obtain the popular news they follow and express their opinions. Studies have been conducted worldwide to capture and analyze data from public social media platforms to determine the real opinions of netizens on policies^27^, such as agenda dynamics on social media^28^, whether a policy is responsive to public opinion^29^, and whether the public opinion process is in accordance with the policy process^30^. Some have attributed the factors influencing public opinions and sentiment to policy regulation at the government level, suggesting that emergency management agency decisions play a role in the inclination of the public toward emotions such as anger and the spread of public opinion^31^. However, there is a lack of corresponding research on how the accumulation and evolution of negative emotions influence policy adjustment.

For long-term public health emergency events, policies must be adjusted according to the specific event to yield optimal strategies^32^. There are many dimensions and indicators that trigger policy adjustments, such as disposal effectiveness^33^, ease of handling, public compliance^34^, and the economy^35^. In this paper, social opinion, which has an impact on policies, is selected as the focus of analysis. Methods for public opinion analysis have been rapidly innovated and developed by combining machine learning, neural networks^36^, Markov chains and other complex mathematical theories as well as computer informatics; coward network and complex network analysis can be used to effectively locate hot spots of public opinion^37^; stream computing methods can be combined with many servers and resources^38^; and similarity analysis approaches for the internet public opinion based on information entropy can cluster and identify hot spots and crisis events in the internet public opinion^39^. Additionally, Markov chains can reflect opinion trends^40^.

We assume that the policy formulation of an emergency management agency is concerned mainly with the effect and cost of the disposal of events and that the feedback on the effect of disposal is based on the level of public satisfaction, which further indicates whether to continue or change the current policy. To explore beyond a supervisory role in today’s informatized China and to provide evidence that public opinion and public sentiment influence policies in the COVID-19 epidemic^41^, rapid policy change is examined as a case study. In this paper, the key time points when the Chinese epidemic greatly changed, as well as the primary affected events and related policies at the end of 2022, are retraced to explore the reasons for the major changes in epidemic prevention policies^42^ and how public opinion influences those policies.

**Figure 1:**
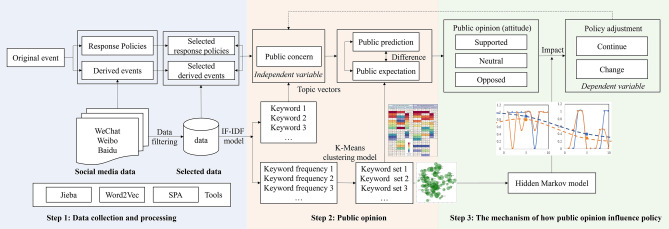
Public opinion analysis framework.

The original event is defined as the earliest event that impacts public safety. When the COVID-19 epidemic emerged as the original event, derived events, which are defined as the public safety event relevant to the original event occurred during its long-term life cycle. The original event has responded policies and derived events. And they own topics in social media such as WeChat, Weibo, and Baidu. We selected the most topical two respond polices and derived events as the selected respond policies and derived events in this paper. Then, we used the IF–IDF model to analyze these data and acquired the top 100 words and their corresponding frequencies. The top 100 words were treated as the public most concerned subjects. Next, we use the K-means model to divide the top 100 words into different groups. And the group themes of selected responded polices and selected derived events were applied to explore the public prediction and public expectation behind public concern, separately. And we analyzed the difference between their prediction and their will. Based on the level of difference, public opinion was divided as suppose, oppose, and neutral. And how public opinion influenced the public polies was explored by the Hidden Markov model. The emergency management agency would determine to continue or change the existing policy. And the new determination would influence the public concern once again as a dynamic process. The research framework is shown in Fig. 1.

## Data and methods

**Figure 2:**
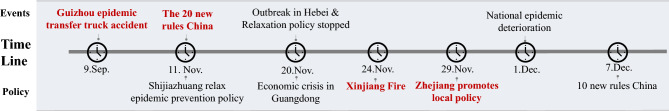
Key events and policies in the last season of 2022.

Derived events and response policies in China spanning from September 1 to December 31, 2022, were compiled and analyzed. On September 19, a tragic incident involving a quarantine transfer vehicle occurred in Guizhou, resulting in the loss of 27 lives. A significant development unfolded on November 11 when China introduced 20 new rules that underscored the implementation of a dynamic zero-COVID policy. Simultaneously, in Shijiazhuang Province, pilot communities began to ease their epidemic control measures. However, the situation changed on November 20, as a severe outbreak in Hebei Province prompted the discontinuation of its role as a pilot community for policy relaxation for epidemic prevention and control. Around the same time, Guangdong Province experienced a grave epidemic situation, triggering an economic crisis. On November 24, a fatal fire in Xinjiang Province claimed 10 lives, generating nationwide concern and a public sentiment crisis^43,44^. By November 29, Zhejiang Province unveiled a policy emphasizing the primacy of public welfare over epidemic prevention, further intensifying the ongoing crisis in public sentiment. As December 1 approached, the epidemic reached a critical juncture in Hebei Province, escalating into a nationwide crisis of epidemic control. In response, China issued 10 new rules on December 7 to stabilize public sentiment and facilitate the country’s reopening. A comprehensive depiction of these events and policies is illustrated in the timeline provided in Fig. 2.

Data harvesting involves utilizing search engines or data sources to target structured data. In this study, we selected the most influential and representative social platforms in China, such as the Sina Weibo, Baidu, and WeChat applets. We collected data within time spans ranging from one week to one month after each event occurred. We encountered a substantial amount of irrelevant content, such as meaningless text and advertisements. We refined event-specific keywords to extract relevant information. We collected the data and chose the two derived events and top two response policies with the highest amount of data. They are the Guizhou epidemic transfer truck accident, the introduction of the new 20 rules in China, the Xinjiang fire incident^45,46^, and the promotion of local epidemic prevention policy in Zhejiang Province.

For the Guizhou epidemic transfer truck accident, we conducted separate searches using keywords such as “Guizhou transit killed” and “Guizhou transit 27 killed”. As the incident had continued impacts, we chose two distinct time periods, namely, November 27 to December 8 and November 23 to December 4, 2022. These searches yielded 716 and 381 data points, respectively, providing valuable insights. Regarding the “20 new rules China”, we employed the keywords “country optimize the 20 new rules China” for our crawl, amassing 1844 data points between November 11 and December 10, 2022. The Xinjiang fire incident, which significantly influenced public opinion and subsequently led to the lifting of lockdown measures, was explored using keywords such as “Xinjiang fire”, “Xinjiang Urumqi epidemic fire 10 deaths”, and “Xinjiang Urumqi epidemic lifting lockdown”. This effort resulted in the acquisition of 1013, 1126, and 1841 data points for three distinct time periods spanning from November 25 to December 8, 2022. Finally, for the topic of “promotion of local epidemic prevention policy in Zhejiang Province”, the search term “Zhejiang Province promoted people in the first place over epidemic prevention policy” was used to gather 622 data points from November 29 to December 6, 2022. Comprehensive information regarding these data points and their specifics can be found in Table 1.

**Table 1:** Data from social media.

Events	Keywords	Items	Time (2022)
Guizhou epidemic transfer truck accident	Guizhou transit accident	716	27 November–8 December
	Guizhou transit 27 killed	381	
Xinjiang fire	Xinjiang fire	1012	25 November–8 December
	Xinjiang Urumqi fire 10 deaths	1126	28 November–8 December
	Xinjiang Urumqi epidemic lifting lockdown	1841	1 November–8 December
Zhejiang promoting local epidemic prevention policy	Zhejiang promoted people first over epidemic prevention	622	29 November–6 December

### Cluster analysis process

**Figure 3:**
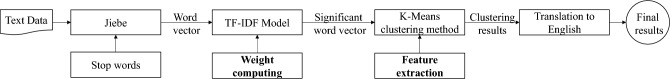
K-means clustering for generating word vectors based on TF–IDF.

In the context of natural language processing, the conversion of lengthy textual information into word vectors is employed to represent the data contained within the text. This allows clustering to be performed by comparing the distances between text vectors, expressing the similarity between word vectors. First, the original text is segmented using the Jieba library. Then, the term frequency-inverse document frequency (TF–IDF) is utilized to calculate the word frequencies within the word vectors. Next, a method involving K-means clustering is employed to select similar words. Finally, the Chinese text is translated into English to obtain the English display results. The processing is conducted as shown in Fig 3.

### Term frequency-invers document frequency model

The term frequency-inverse document frequency (IF–IDF) model is a common weighting technique used in information retrieval and text mining. The word frequency (IF) is calculated first. The word frequency is normalized by dividing the number of times a word appears in an article by the total number of words in the article to facilitate comparisons between articles of different lengths, as shown in Eq. (1).

1 $${\text{IF}}_{i} = \frac{{f_{i} }}{{\mathop \sum \nolimits_{i = 1}^{n} f_{i} }},$$

A weight needs to be assigned to each word based on the word frequency extraction. This weight is called the inverse document frequency (IDF), and its size is inversely proportional to how common a word is. The IDF is calculated for a word based on the corpus simulating the linguistic environment; the more common the word is, the larger the denominator and the closer the IDF is to 0. To avoid situations in which not all documents contain the word, 1 is added into the denominator, as shown in Eq. (2).

2 $${\text{IDF}}_{i} = \log \left( {\frac{{w_{i} }}{{\mathop \sum \nolimits_{i = 1}^{n} w_{i} + 1}}} \right),$$

The IF–IDF is obtained by multiplying the resulting word frequency (TF) by the inverse document frequency (IDF). It is proportional to the number of times a word appears in the document and inversely proportional to the number of times the word appears in the language. The more important a word is to the text, the greater its IF–IDF value. The words that come first are therefore the keywords for the information in the article, as shown in Eq. (3).

3 $$\left( {{\text{ TF}} - {\text{IDF}}} \right)_{i} = {\text{IF}}_{i} \times {\text{IDF}}_{i} ,$$

### K-means clustering model

Clustering involves the unsupervised categorization of data based on inherent relationships without prior knowledge of sample labels. Multiple forms of clustering exist, including hierarchical, divisional, density, grid, and fuzzy clustering. In our study, we opted for the K-means method within the divisional clustering framework, which is renowned as a classic approach. This method uses an iterative procedure to identify $k$ clusters, aiming to minimize the loss function corresponding to the resultant clustering. The iterative step is repeated until process $J$ converges. The loss function is mathematically defined as Eq. (1), which drives the clustering refinement.

4 $$J\left( {c,p} \right) = min\sum\nolimits_{I = 1}^{N} {\left\| {x_{i} - p_{{c_{i} }} } \right\|^{2} }$$

${x}_{i}$ represents sample $i$; ${c}_{i}$ represents the cluster to which ${x}_{i}$ belongs; ${p}_{{c}_{i}}$ represents the centroid corresponding to the cluster; $N$ is the total number of samples.

### Hidden Markov model

Public opinion data are analyzed to obtain key indicators representing public expectation and policy adjustment through an unsupervised machine learning approach based on the hidden Markov model. The Markov model is a stochastic process in which the variables depend on the states preceding the sequence. This process is a dual stochastic process of model state transitions and observable event randomness in a particular state.

The model is determined by the initial probability distribution, the state transfer probability distribution and the observation probability distribution, the Markov model $\varphi =\left(A, B,\pi \right)$.${\text{Q}}$ is the set of all possible states; and P is the set of all possible observations. $n$ is the number of possible states, $m$ is the number of observations, $I$ is the observation sequence of length $T$, and O is the corresponding observation sequence. $A$ is the state transfer matrix, and ${a}_{ij} i$ s the probability of transferring to state ${q}_{j}$ at moment $t+1$ under the condition that moment $t$ is in state ${{\text{q}}}_{i}$. $B$ is the observation matrix. π is the initial state vector. The system of formulas is shown in Eq. (5).

5 $$\begin{aligned} & Q = \left\{ {q_{1} ,q_{2} , \ldots ,q_{n} } \right\},\,P \, = \left\{ {p_{1} ,p_{2} , \ldots ,p_{m} } \right\}, \\ & I = \left\{ {i_{1} ,i_{2} , \ldots ,i_{T} } \right\},\,O = \left\{ {o_{1} ,o_{2} , \ldots ,o_{T} } \right\}, \\ & A = \left[ {a_{ij} } \right]_{n \times n} , \\ & a_{ij} = P\left( {i_{t + 1} = q_{j} |i_{t} = q_{i} } \right), \quad i,j = 1,2, \ldots ,n, \\ & B = \left[ {b_{j} \left( k \right)} \right]_{n \times m} , \\ & b_{j} \left( k \right) = P\left( {o_{t + 1} = v_{k} |i_{t} = q_{j} } \right),\quad k = 1,2 \ldots , m; \quad j = 1,2, \ldots ,n, \\ & \pi_{i} = P\left( {i_{1} = q_{i} } \right),\quad i = 1,2, \ldots ,n, \\ \end{aligned}$$

## Results

### Core elements and dimensions in the reasoning process of public opinion

We have developed a systematic approach to understanding public opinion. This process involves five key elements: the original event, derived events, public concern, public prediction, and public expectation. These elements serve as the foundation for our analysis. Specifically, we focus on four dimensions: the original event, derived events, public prediction, and public expectation.

In this paper, our initial approach involves analyzing the text of four selected events utilizing the Word2Vec method. This method allows us to extract feature subsets from the original dataset while filtering out irrelevant elements such as dates using stop words. We then identify the top 100 keywords. Subsequently, to effectively capture these dimensions, we aim to identify corresponding indicators. To achieve this, we employ the K-means text clustering method on the top 100 keywords. This process yields sets of word vectors, where vectors in proximity within vector space share similar contextual meanings, while distant vectors signify differing meanings. Then the group themes are divided into different dimensions. For original and derived event, we referred to the 5 M model, originally developed by T.P. Wright at Cornell University, serves as a risk management framework for safety. This model is rooted in the concept of the man–machine-environment triad. It incorporates five fundamental elements: human factors, machinery, management practices, environmental factors, and mission objectives. For public prediction and expectation, we draw upon expertise knowledge. Results are shown in Table 2.

**Table 2:** Dimensions and indicator construction of public concern.

Dimensions	Indicators	Example of the top 100 words
Original event	Epidemic name	COVID-19, epidemic, pneumonia
	Infections	Cases, positive
	Virus	Asymptomatic
	Status	Death, virus parameters
	Policies	Close loop, initial screening
Derived event	Event name	Fire, transfer
	People	Experts, out of province
	Materials	Wire board
	Environment	Xinjiang, time
	Response	Detection, prevention
	Consequence	Injured, death
Public predication	Residence	Mass, public
	Socially vulnerable groups	The elderly
	Measures	Government, current
	Projections	According to, other provinces
	Environment	Community, neighborhood
	Service	Designed hospitals, medical observation
	Livelihood	High risk (area), local
	Management	Recovery, homestay
Public expectation	Object	Government, country
	Condition	Public health, economy
	Requirement	Optimization, development
	Desires	Release, scientific

### Public concern

To investigate the primary areas of public concern, we selected the top twenty words from the previously identified top 100 words that encapsulate a wide range of public concerns while maintaining their content’s significance. Subsequently, we conducted a thorough analysis of public concerns within the framework of the dimensions we have identified.

### Derived events

**Figure 4:**
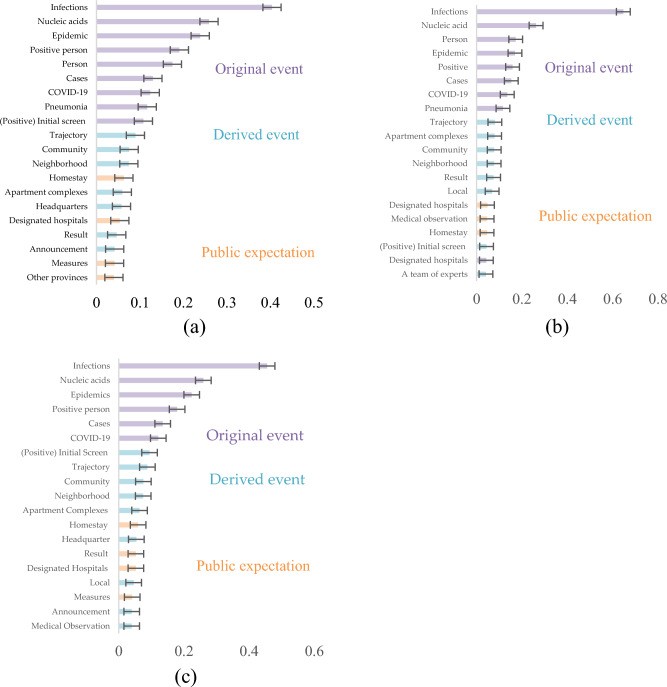
The top 20 words of public concern of Guizhou epidemic transfer truck accident. (**a**) The top 20 words of public concern of group 1: result of key words: Guizhou transit accident; (**b**) The top 20 words of public concern of group 2: result of key words: Guizhou transit 27 killed; (**c**) The top 20 words of public concern of total: result of key words: Guizhou epidemic transfer truck accident.

In the context of the Guizhou epidemic transfer truck accident, Fig. 4c depicts a notable level of concern regarding infection, with a value of 0.489, followed by nucleic acids at 0.260, and epidemic concerns at 0.214. Figure 4a,b were generated based on search queries related to “Guizhou transit accident” and “Guizhou transit 27 killed,” respectively. A discernible difference between these figures is observed, where individuals searching for accidents exhibit a greater focus on the original event, while those exploring the death are more inclined towards details pertaining to derived events.

**Figure 5:**
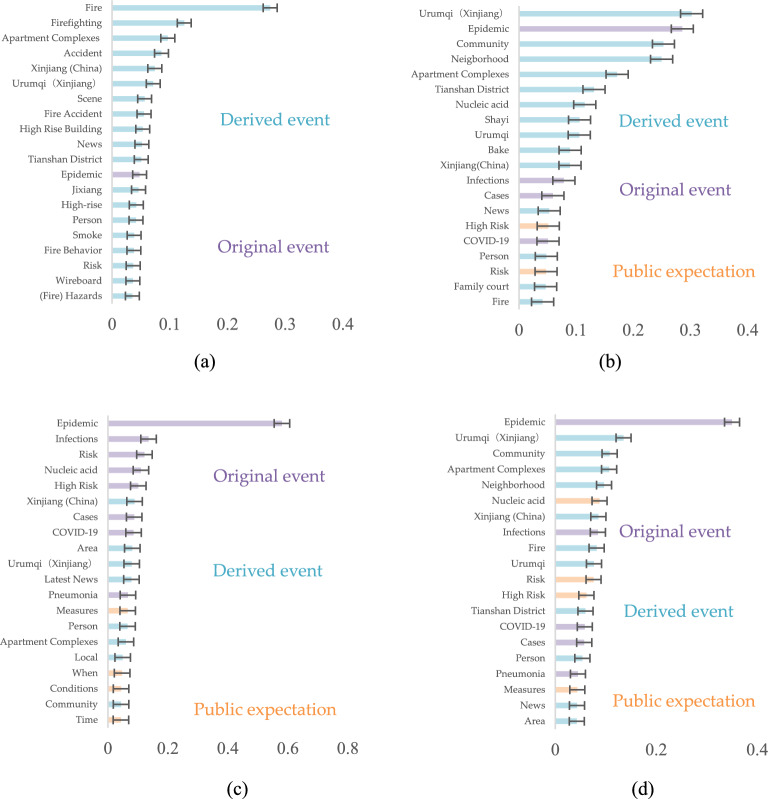
The top 20 words of public concern of Xinjiang fire accident. (**a**) The top 20 words of public concern of group 1: result of key words: Xinjiang fire; (**b**) The top 20 words of public concern of group 2: result of key words: Xinjiang Urumqi fire 10 deaths; (**c**)The top 20 words of public concern of group 3: result of key words: Xinjiang Urumqi epidemic lifting lockdown; (**d**) The top 20 words of public concern of total: Result of keywords: Xinjiang province fire accident.

Xinjiang fire, Xinjiang Urumqi fire 10 deaths, and Xinjiang Urumqi lifting lockdown are obtained as three keywords, respectively, for the results in Fig. 5a–c. The above search results are analyzed together to obtain Fig. 5d. Overall, the attention to the derived event details and the original event is remarkable in the Xinjiang fire, such as its reason (wire board), location (Jiixiang Yuan neighborhood in Tianshan District, Urumqi, Xinjiang), building type (high rise building), fire behavior, circumstance (smoke), firefighting results, and latest news. Considering there is a portion of the public that feels response policy is excessive and is dissatisfied with the prolonged district lockdown. The derived event becomes an outlet for public opinion about the policies responding to the original event, suggesting that prolonged and stringent prevention policies can trigger public emotional repression and tension that needs to be released. The public express their expectations for changes in lockdown measures by searching for more news about the polices.

However, the results show differences depending on the search terms. People who search for the Xinjiang fire are extremely concerned with details of derived event while people who search for deaths are more concerned with original event. People who search for the lockdown display a moderate level of concern, accompanied by expressions of lifting lockdown. Public expectations are expressed in the concern of time (when) and the conditions (condition) of lifting lockdown.

***Response policies***

**Figure 6:**
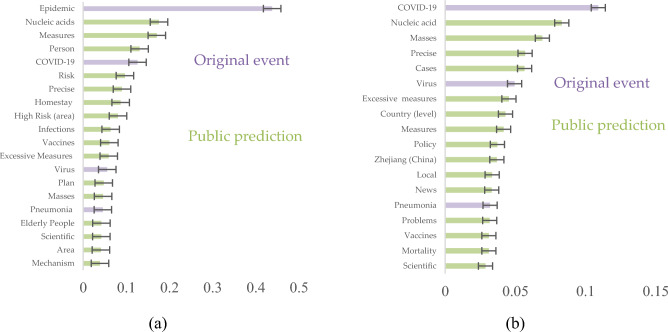
The top 20 words of responded policies. (**a**) The top 20 words of the 20 new rules China. (**b**) The top 20 words of Zhejiang promoting local policies.

For the 20 new rules China, the results are shown in Fig. 6a, indicating that the public is more interested in public prediction, they predict whether a new scientific policy can be proposed on a national scale to solve the existing problems. The concerns about specific measures such as nucleic acids with a result of 0.176. Because at that time, nucleic acid testing was required every two days, which was time consuming and affected daily life, so there was great interest about whether new policy would solve the issue. Claims about precise prevention and control, homestay, vaccines, and measures were also a large part of the concerns. For New Policies in Zhejiang, Fig. 6b illustrates a notable trend that the public displays higher concern for predictive aspects while displaying comparatively diminished concern for the original event. They anticipate that local and national governments will introduce policies to address pressing concerns, such as the rising number of cases, mutated viruses, vaccine effectiveness, nucleic acid testing, and precise control measures.

### Public expectation and public prediction

We further examined the selected derived events and response policies related to the original event. These are initially discussed as a whole and subsequently categorized into two groups for separate study. The Guizhou epidemic transfer truck accident and Xinjiang fire are classified under the “affected event” category, while the “Zhejiang promotes people-first epidemic prevention” policy and the “20 new rules in China” are in the “major policy” category.

**Figure 7:**
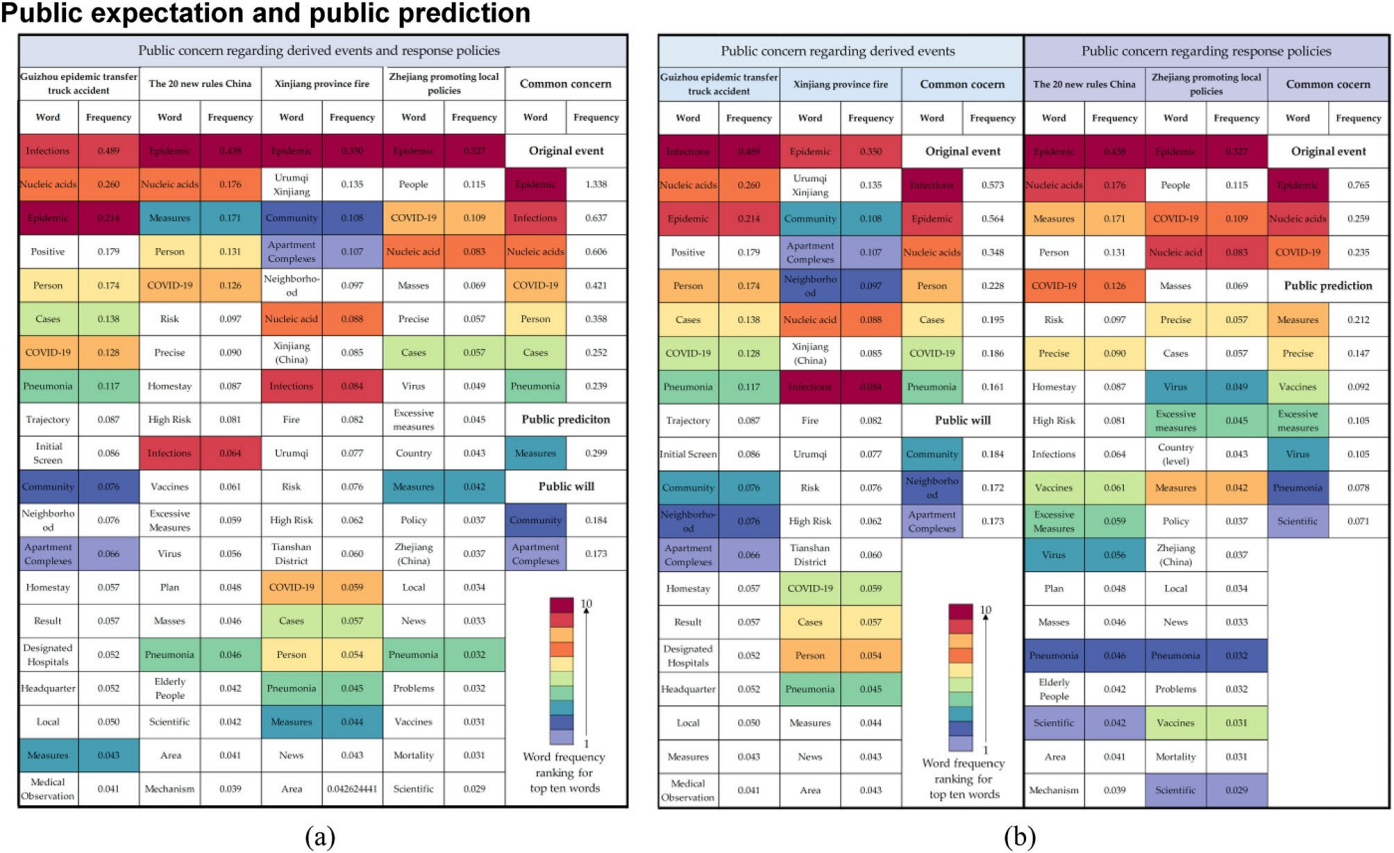
Public expectations and public predictions behind the public concern and (**a**) Common concerns to affected events and original event policies. (**b**) Different concerns in affected events and original event policies.

Figure 7a illustrates the common concerns in these events are the original event, public expectation, and public prediction. Notably, there is a large amount of data about the derived events, however, the results show that the public is concerned about key information about the epidemic situation and searches for information about the policy changes. Therefore, high-frequency keywords represent the public expectation.

Differences in the public concern for derived events and response policies of the original event in the epidemiological background are shown in Fig. 7b. For derived events, a substantial portion of public concern revolves around aspects of the original event, such as infections, the overall epidemic situation, and nucleic acid testing (a critical epidemic prevention measure in China). Moreover, public expectations pivot towards prospective enhancements within their immediate living environments, evinced by the prominence of terms such as communities, neighborhoods, and apartment complexes. This emphasis underscores the community-centric ethos inherent in epidemic prevention policies during the specified period.

### Public opinion evolution and mechanisms for influencing policy adjustments

As the epidemic progressed, public expectations evolved. Initially, the public sought stringent epidemic response policies integrated into their daily lives. However, over time, there was a growing preference for easing excessively strict measures like home stays and lockdowns. Public satisfaction with current policies tends to influence their concern towards response policies, with adherence to existing policies and anticipation of future ones. Conversely, dissatisfaction leads to disregard for response policies and decreased concern data. Public concern shifts towards derived events believed to be influenced by unreasonable policies, resulting in increased expressions of complaints and expectations.

We analyze public concerns across various dimensions of the event and suggest that the level of concern regarding specific measures correlates positively with overall policy satisfaction. Predicting epidemic trajectories is challenging due to their prolonged nature and evolving dynamics, compounded by virus mutations. China has grappled with the Delta variant over the past two years, characterized by average transmission but high mortality rates. Stringent response policies in China effectively controlled domestic infection and mortality rates, albeit with mobility and economic constraints. The emergence of the highly transmissible yet less lethal Omicron variant has led to a growing demand for relaxed epidemic policies.

**Figure 8:**
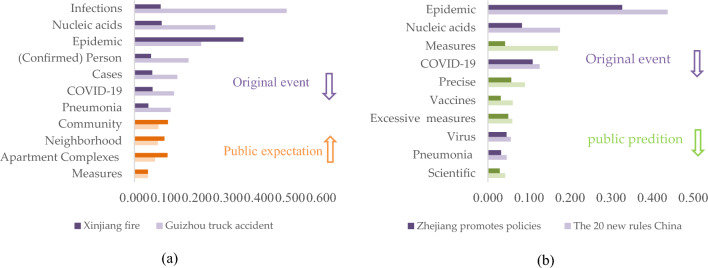
Changes in public opinion. (**a**) Public opinion changes at derived events. (**b**) Public opinion changes at responded policies.

Figure 8 displays the top ten words and their corresponding frequencies, as depicted in Fig. 7b. Analyzing shifts in public attention reveals evolving priorities according to results in Fig. 8, with increased focus on specific details like infections and nucleic acid testing, and decreased emphasis on original events and public prediction. This indicates anticipation for policy relaxation due to public dissatisfaction. Public concern towards original events serves as an indicator of support for current policies, with satisfaction rates of 73% after the Guizhou transit accident and 35% after the Xinjiang fire.

**Figure 9:**
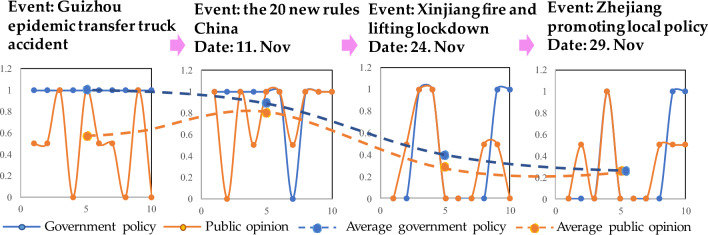
The process control of reaching consensus on public opinion and policy.

For the policies in response to the epidemic, the 20 new rules in China and local improvement policies in Zhejiang Province have been great catalysts for the sudden move from closed to open policy in China. The results are shown in Fig. 9. Common concerns about the 20 new rules in China and local improvement policies in Zhejiang Province exhibited a downward trend in all aspects. The greatest decline in concern was for specific measures such as nucleic acid testing and vaccination. At this point, the public is no longer enthusiastic about improving epidemic prevention measures. The distribution of concern data in Zhejiang shows that the public is turning to local policies in Zhejiang and expecting new policy changes nationwide. This shows that at this time, the public’s reaction to the policy is less intense, and negative rhetoric, similar to transmission prevention and control, is no longer as much of a concern. This shows that the public’s reaction to the policy is not very intense and that the level of attention given to negative statements such as excessive measures is not very high. The satisfaction level was 37% for the 20 new rules in China and 29% for the Zhejiang promotion of local policies.

There were shifts in public attention and changes in satisfaction levels throughout the original event. In this paper, the entire process of triggering a policy shift is described by tracking such changes. The HMM is applied to explore public opinion and emergency management agency policy in the short-term to reach a consensus. The hypothesis is that the set of governmental states Q is considered to encompass two states of policy continuation and change, denoted by 1 and 0, respectively. The corresponding set of the public expectation observations P encompasses the three states of support, neutrality and opposition, denoted by 1, 0.5 and 0, respectively. Based on the real situation at that time, the Chinese emergency management agency had no thoughts of changing its policy. The state probability vector π of the initial node is 1 for continuing and 0 for changing. As events occur, public attention to affected events and new policies shows a state transfer matrix $A$ set to 0.7 for the probability of maintaining the original satisfied state and 0.3 for the probability of switching to the unsatisfied state. Similarly, the probability of maintaining the original unsatisfied state is 0.7, and the probability of switching to the satisfied state is 0.3 according to the policies of the original event. For policy satisfaction, the probability matrix $B$ of public opinion data observed through the public platform indicates a 0.6 probability of support, 0.3 probability of neutrality, and 0.1 probability of opposition; in the case of dissatisfaction, there is a 0.1 probability of support, 0.3 probability of neutrality, and 0.6 probability of opposition according to the statistics on public attention given to the affected events. The simulations are performed 10 times, and the observed averages are calculated. The changes in the will of emergency management agencies and the attitude of public opinion over time are given in Fig. 9.

$$\begin{aligned} & {\text{Q }} = \, \left( {{\text{Continue}},{\text{ Change}}} \right) \, = \, \left( {{1},0} \right); \\ & {\text{P }} = \, \left( {{\text{Support}},{\text{ Neutral}},{\text{ Opposed}}} \right) \, = \, \left( {{1},0.{5},0} \right); \\ & {\uppi } = \left( {{\text{Continue}},{\text{ Change}}} \right) \, = \, \left( {{1},0} \right); \\ & {\text{A }} = \left( {\begin{array}{*{20}l} {{\text{Continue}}|{\text{ Continue }}} \hfill & {\quad {\text{Continue}}|{\text{ Change}}} \hfill \\ {{\text{Change}}|{\text{ Continue}}} \hfill & {\quad {\text{Change}}|{\text{ Change}}} \hfill \\ \end{array} } \right) = \left( {\begin{array}{*{20}l} {0.7} \hfill & {\quad 0.3} \hfill \\ {0.3} \hfill & {\quad 0.7} \hfill \\ \end{array} } \right); \\ & {\text{B}} = \left( {\begin{array}{*{20}l} {{\text{Continue}}|{\text{Support}}} \hfill & {\quad {\text{Continue}}|{\text{Neutral}}} \hfill & {\quad {\text{Continue}}|{\text{Opposed}}} \hfill \\ {{\text{Change}}|{\text{Support}}} \hfill & {\quad {\text{Change}}|{\text{Neutral}}} \hfill & {\quad {\text{Change}}|{\text{Opposed}}} \hfill \\ \end{array} } \right) = \left( {\begin{array}{*{20}l} {0.6} \hfill & {\quad 0.3} \hfill & {\quad 0.1} \hfill \\ {0.1} \hfill & {\quad 0.3} \hfill & {\quad 0.6} \hfill \\ \end{array} } \right) \\ \end{aligned}$$

The results show that at the time of the Guizhou transit accident, the average public opinion state was support, and the emergency management agency opinion was to maintain the policy. The public opinion synthesis indicates a neutral attitude, with a 50% support rate. The emergency management agency was willing to continue the policy. During the time leading to September 2022, when the 20 new rules were released in China, the public support increased to 80%, indicating that the public wanted the new policy to be enacted. The emergency management agency tends to change its policy, reflected by a slight intention to change. After the Xinjiang fire, public opposition rose to 35%. Emergency management agency policies were issued primarily in response to public safety events to achieve satisfactory outcomes for the public. During the evolution of a long-term public health event, the public expects the policy to be updated as the original event situation changes. Maintaining the original policy can lead to increased public opinion dissatisfaction with the policy. After the release of local policies in Zhejiang Province, public support slowly dropped to 30%. The emergency management agency will continue the policy decreased and the will to adjust the policy rose to a certain level, consistent with the public expectation. Continuous changes in the will of the public caused the emergency management agency to consider the public expectation, triggering a policy change in response to public opinion. This facilitated the subsequent promulgation of the new 10 rules in China and the rapid opening shift. The evolution of the process of reaching consensus on public opinion and policy is shown in Fig. 9.

## Discussion

The findings from our study indicate a changing pattern in public concern regarding derived events and response policies. Initially, the public is concerned about derived events and expresses expectations regarding the original event. Meanwhile, they also show concern for existing response policies and attempt to predict future policy developments. Our research suggests a correlation between increased public concern and stronger expectations or predictions. When the public perceives satisfaction with current policies, their attention shifts towards existing policies. However, if they lose confidence in the emergency management agency, they prioritize concern for derived events and anticipate new policies. This shift in attention highlights the proactive nature of the public in monitoring policies in real time. Moreover, our analysis reveals that when expectations are met, there is a more positive attitude towards the policy. Notably, our approach considers the dynamic evolution and persistence of public opinion on event concerns, a factor that has received less attention in previous studies.

Previous studies on policies focused mainly on the government’s perspective^47^, which was concerned primarily with the effect of disposing of things, and public opinion was not considered one of the factors influencing policies in China but rather a manifestation of government guidance^48^. However, in today’s information society, public opinion is not only guided by the government but is also increasingly spontaneous^49^; thus, public opinion becomes the main factor triggering emergency management agency policy adjustment when it reaches a certain level, which may differ from the present case in other cases.

In the realm of epidemic management, variations in systemic elements play a pivotal role. Our findings suggest that emergency management agencies should incorporate more intricate indicators into their decision-making processes regarding the continuity or modification of existing policies. Given the protracted nature of public events, individuals will persistently scrutinize both the original event and its derived consequences, shaping their beliefs and adherence to response measures. Consequently, emergency management agencies must diligently monitor public sentiment and approach derived events with heightened caution. It is imperative to acknowledge and comprehend the complex interplay of systemic elements. Epidemic risks are intertwined with factors such as public opinion, political dynamics, economic fluctuations, and technological considerations.

A management approach centered on silence can impede economic progress and incite political instability, while an open-oriented strategy may precipitate medical crises and public hysteria. Public sentiment serves as a pivotal determinant in the deliberations of emergency management agencies regarding policy adjustments, particularly in the face of negative sentiments like widespread anger. Hence, conducting a thorough assessment of the mechanisms through which public opinion influences policy implementation is paramount for safeguarding national security.

## Conclusion

We employ the IF–IDF model, K-means model, and Hidden Markov Model (HMM) to analyze the influence of public opinion on adjustments to public health policies. Our approach involves examining the reasoning process of public opinion based on a knowledge base. Public concern regarding response policies and derived events serves as input and forms the foundation for exploring public predictions and expectations within our reasoning model. Through a case study, we demonstrate the cumulative impact of derived events related to the original event on public expectations during the original event period. Our findings suggest that public opinion plays a significant role in shaping current COVID-19 prevention policies in China. There exists a feedback moderating effect on policies, potentially leading to adjustments following implementation. Furthermore, our work provides insights for studying changes in public health risk management policies.

In this paper, we focus solely on the impact of individual events on changes in public expectation. Future research should consider cumulative changes in public expectation over time. Additionally, additional dimensions of public opinion should be explored to better reflect changes in public expectation. Detailed portrayals of the cumulative effect of events on public expectation can be achieved through more sophisticated quantitative models. Subsequent research efforts should aim to establish more realistic settings for policy parameters and hidden Markov models based on epidemic.

## Data Availability

The datasets analysed during the current study are not publicly available due to privacy or ethical restrictions, but are available from the corresponding author on reasonable request.
